# Bangladesh Cerebral Palsy Register (BCPR): a pilot study to develop a national cerebral palsy (CP) register with surveillance of children for CP

**DOI:** 10.1186/s12883-015-0427-9

**Published:** 2015-09-25

**Authors:** Gulam Khandaker, Hayley Smithers-Sheedy, Johurul Islam, Monzurul Alam, Jenny Jung, Iona Novak, Robert Booy, Cheryl Jones, Nadia Badawi, Mohammad Muhit

**Affiliations:** The Children’s Hospital at Westmead (Clinical School), The University of Sydney, Locked Bag 4001, Westmead, NSW 2145 Australia; Cerebral Palsy Alliance Research Institute, The University of Sydney, Westmead, Australia; Child Sight Foundation (CSF), House 9, Flat A1& B3, Road 2/1, Banani, Dhaka 1213 Bangladesh; School of Public Health, Sydney Medical School, The University of Sydney, Westmead, Australia; National Centre for Immunisation Research and Surveillance (NCIRS) and The University of Sydney, Westmead, Australia; The Children’s Hospital at Westmead and the Marie Bashir Institute, University of Sydney, Westmead, Australia; Cerebral Palsy Alliance Research Institute and the Children’s Hospital at Westmead, Westmead, Australia; Child Sight Foundation (CSF) and University of South Asia, House 9, Flat A1& B3, Road 2/1, Banani, Dhaka 1213 Bangladesh

**Keywords:** Cerebral palsy, Childhood disability, Register, Bangladesh

## Abstract

**Background:**

The causes and pathogenesis of cerebral palsy (CP) are all poorly understood, particularly in low- and middle-income countries (LMIC). There are gaps in knowledge about CP in Bangladesh, especially in the spheres of epidemiological research, intervention and service utilization. In high-income countries CP registers have made substantial contributions to our understanding of CP. In this paper, we describe a pilot study protocol to develop, implement, and evaluate a CP population register in Bangladesh (i.e., Bangladesh Cerebral Palsy Register - BCPR) to facilitate studies on prevalence, severity, aetiology, associated impairments and risk factors for CP.

**Methods/Design:**

The BCPR will utilise a modified version of the Australian Cerebral Palsy Register (ACPR) on a secured web-based platform hosted by the Cerebral Palsy Alliance Research Institute, Australia. A standard BCPR record form (i.e., data collection form) has been developed in consultation with local and international experts. Using this form, the BPCR will capture information about maternal health, birth history and the nature of disability in all children with CP aged <18 years. The pilot will be conducted in the Shahjadpur sub-district of Sirajgonj district in the northern part of Bangladesh. There are 296 villages in Shahjadpur, a total population of 561,076 (child population ~ 226,114), an estimated 70,998 households and 12,117 live births per annum. Children with CP will be identified by using the community based Key Informants Method (KIM). Data from the completed BPCR record together with details of assessment by a research physician will be entered into an online data repository.

**Discussion:**

Once implemented, BCPR will be, to the best of our knowledge, the first formalised CP register from a LMIC. Establishment of the BCPR will enable estimates of prevalence; facilitate clinical surveillance and promote research to improve the care of individuals with CP in Bangladesh.

## Background

Cerebral palsy (CP) is one of the major causes of childhood disability with an estimated global incidence between 2 and 3 per 1000 live births [1]. Although it is estimated that CP is 5 to 10 times more common in underprivileged parts of the world, the exact burden is unknown in most low- and middle-income countries (LMIC) [2].

Globally, 85 % of children with disabilities live in developing countries, but less than 5 % receive rehabilitation services [3]. It is hypothesised that the causes of CP in developing countries are different from those in high resource countries, although this has to date not been investigated deeply. A recent study from Nigeria found that birth asphyxia (39.0 %), bilirubin encephalopathy (24.4 %) and post-infectious brain damage (18.3 %) were the major causes of CP [4]. Bangladesh is a densely populated country in South Asia with an estimated 2.6 million children living with severe disabilities but only 1500 children in the country have access to education in special schools sponsored by the Government [5]. In a recent pilot study on the infectious causes of childhood disabilities, 859 children with severe physical impairment were identified from a rural sub-district (i.e., Shahjadpur) of Bangladesh of which 417 (48.5 %) had CP [6]. Over half of those children (57 %) had never received any rehabilitative support or services. Only 21.1 % (182) of those children were attending regular school and just 0.2 % (2) were attending special schools [6]. Another study using Key Informant Method (KIM) reported an estimated prevalence of CP up to 3.7/1000 children in Bangladesh (95 % CI 3.5–3.9) [7]. According to this conservative estimate, there are ~ 260,000 children with CP in Bangladesh.

The causes and pathogenesis of CP are not well understood, particularly in developing countries where causality and rates of survival may differ [8]. There are potential gaps in knowledge of CP in Bangladesh, especially in the spheres of epidemiological research, intervention and service utilization. With little known about CP in Bangladesh, researchers, the government and service providers are limited in their capacity to quantify current and future resource allocation needs or to identify preventive strategies. CP research in developing countries is further compromised by the lack of a representative population sample. Population data are required to reduce potential inaccuracy in research conclusions based on non-representative groups of children with CP (e.g., hospital based research); and for ensuring that strategies developed are applicable to people living with CP in similar settings.

In high-income countries, substantial knowledge on the prevalence, risk factors, distribution, frequency, and severity of CP has been explored and defined through CP registries [9]. A ‘CP register’ is a confidential research database which collects information about a population of people with CP. The main aim for CP registers generally is to monitor the incidence and prevalence of cerebral palsy as well as co-morbidites, and to gain further understanding about the aetiologies of CP to design and evaluate preventative strategies as well as monitor service delivery trends [9].

CP registers have been established in Europe and Australia for more than 30 years [9]. In 1999, Surveillance of Cerebral Palsy in Europe (SCPE) was established combining CP registers and surveys from 9 European countries to form a framework for collaborative research. Western Australia’s CP register was the first Australian register established in 1979, and subsequently other state/territory CP registers were established. In 2007, a nation-wide CP register, the Australian Cerebral Palsy Register (ACPR) was initiated. As part of this collaboration, de-identified data is contributed from all 7 Australian state/territory CP registries to one common database [9, 10]. Over the last 3 decades, the Australian and European CP registers have generated an enormous amount of original data on the incidence, prevalence, aetiologies and risk factors for CP in high-income countries. Moreover, large scale intervention trials have been conducted using the CP registers as sampling frame [10]. However, till now there is no CP registers established in LMIC.

In this paper, we describe a pilot study protocol to develop, implement then evaluate a CP population register in Bangladesh following the existing infrastructure of the ACPR. Once established BCPR will work as a platform for a National Cerebral Palsy Register in Bangladesh to facilitate clinical surveillance and promote research to improve the care of individuals with CP and will be a model for other LMIC.

## Methods

### Aims and objectives

The aim of this pilot study is to develop a platform for national CP register in Bangladesh to facilitate studies on prevalence, severity, aetiology, associated impairments and risk factors for CP in Bangladesh.

Our specific objectives are:

1. To establish a platform for national CP register in Bangladesh utilizing available infrastructure from the ACPR.
2. Determine the prevalence and profile of children with cerebral palsy in Bangladesh.
3. Understand the aetiology and risk factors of CP in Bangladesh.
4. Identify opportunities for prevention e.g., improving maternal hygiene, immunisation programs.
5. Use the CP Register as a sampling frame for future research e.g., to trial and evaluate cost-effective intervention strategies which promote functional rehabilitation and limit associated or secondary impairments.
6. Evaluate BCPR pilot study as a model to develop platform for national CP register in Bangladesh and other LMIC.

### Hypothesis/research question

We hypothesize that the BCPR will enable accurate estimates of disease burden (including incidence, prevalence); facilitate clinical surveillance; and promote international collaboration to research the translation of contemporaneous evidence-based care into affordable service delivery models to improve the management for children’s with CP in Bangladesh.

### Overview of study design

#### Establishment of the Bangladesh Cerebral Palsy Register (BCPR)

BCPR will capture information about maternal health, birth history and the nature of the resulting disability of all children with cerebral palsy aged <18 years in the study area (i.e., Shahjadpur). A modified version of the ACPR record form will be used to collect information from parents/carers of children with CP. The BCPR record form will be completed by a medical practitioner and a detailed clinical assessment will be performed prior to collecting information for the BCPR record form.

The initial set up phase will seek to establish the processes necessary for ongoing data collection and secure representative register management. These processes will include:

1. Obtaining ethical approval from participating services and institutions to collect data.
2. The establishment and convening of a CP Register Advisory Board (i.e., BCPR Advisory Board).
3. The engagement of software engineers to develop the BCPR infrastructure through the Cerebral Palsy Alliance Research Institute.
4. Recruitment of a register staff member to operationalize the register and commence data collection procedures using the Key Informants Method (KIM) in the study area.
5. The dissemination of register procedures to members of relevant professions (e.g., health professionals, physiotherapist and occupational therapist) and people with CP who will be invited to notify ‘CP records’ to the register.

### Case definition

The BCPR will adopt the approach used by the SCPE and the ACPR which allows the use of any definition for CP that includes the following five key elements common to the published definitions [11–16].

Cerebral palsy:

1. Is an umbrella term for a group of disorders,
2. Is a condition that is permanent but not unchanging,
3. Involves a disorder of movement and/or posture and of motor function,
4. Is due to a non-progressive interference, lesion, or abnormality, and
5. The interference, lesion, or abnormality originates in the immature brain.

### Inclusion/exclusion criteria

In order to be included in the BCPR dataset, a case must fulfil the criteria contained in the five definitional elements. In children aged <5 years their inclusion in the CP register will be reviewed when the individual reaches 5 years of age. In the event that new information becomes available a case entry may be updated, which may involve inclusion or exclusion from the CP register [15, 16].

### Study location and population

**Fig. 1:**
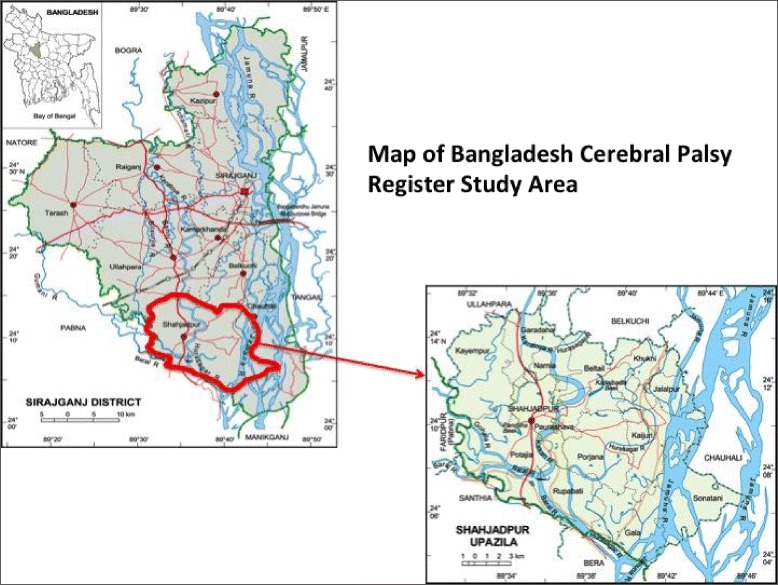
Study area of the BCPR (sub-district Shahjadpur) located in the North of Bangladesh [18]

The pilot study will be conducted in the Shahjadpur sub-district of Sirajgonj district in northern part of Bangladesh. There are 296 villages in the study area with a total population of 561,076 (child population ~ 226,114) and an estimated 70,998 households, and 12,117 live births per annum. (Fig. 1) [17, 18].

The underlying goal of BCPR is to develop as a population CP register. In line with this principle we would aim to conduct our pilot study activities in a defined geographical area (i.e., Shahjadpur sub-district) with known population denominator. We would also keep an up-to-date record of the population denominator of the study catchment area including total and child population, number of live births, perinatal mortality rates, and information on population migration (when available) from local councils and statistical department. If any of this information is not available, we will use national representative census data.

### Study participants and sources and means of recruitment

#### Existing cohort

Based on a recent study, the estimated prevalence of cerebral palsy in Bangladesh is 3.7/1000 children [7], which is 1.5 times higher than Australia or Europe. Accordingly, there are around 700 children (95 % CI 661–737) with CP in Shahjadpur. We previously conducted a pilot study on infectious causes of childhood disability in the study area and identified 417 children with CP (aged 2–17 years) [6]. From the pilot study, we have obtained contact information of all children identified with CP, and would be able to retrace those children to include into the BCPR. For newly diagnosed CP cases we will also utilize our existing network of key informants to identify disabled children from the community.

#### Identification of children with CP from communities using key informant’s method (KIM)

KIM is a novel method for identifying disabled children in the community. It involves training local volunteers to act as key informants (KIs). KIs are people who live and/or work in their local community, who have a social role through their vocation, and who are, therefore, likely to know the local context as well as the people about whom information is being sought. [19, 20] Our collaborating organisation in Bangladesh (Child Sight Foundation – CSF, Bangladesh) has already trained over 240 KIs (approximately one per village) in the study area (sub-district of Shahjadpur) and over 23,000 KIs nationally. For this study, two trained community mobilisers (CMs) will be working with the task to identify new KIs in the study area. These KIs will be given day-long structured training on CP and group sessions on awareness raising and disability-specific information using flip chart illustrations and role play with guidance led by the CMs. After the initial training, KIs will be given 4–6 weeks to find and list children with specific physical impairments in their local community and will be informed of the date and venue of the KIM medical assessment “camps”. The existing network of KIs will also be utilised to identify children with CP in the study area. The children/families identified by the KIs will be offered a consultation at the medical assessment camp, to provide an opportunity for diagnosis, treatment and referral of their child.

#### Medical assessment

Medical assessment camps will be conducted on a monthly basis in the study area and will comprise a paediatrician, physiotherapist and a counsellor. Team members will be trained in using BCPR record form (i.e., data collection form). All the children referred by the KIs will be examined to assess their physical condition and to determine if they meet the inclusion criteria for the CP register. Families will be provided with appropriate advice, information and counselling and referral services. The BCPR record form will be completed onsite by the attending paediatrician.

### Other sources of recruitment

We plan on extending BCPR activities to adjacent sub-districts and other parts of the country following implementation of this pilot register (Table 1). We will collect information from multiple sources including: hospitals, rehabilitation centers, other NGOs working with children with CP to contribute to BCPR by registering children with CP through the online and/or paper based registration system. However, data would be reported separately for those with known population denominator (i.e., representing a population register).

**Table 1:** Proposed BCPR activity

	BCPR pilot phase 2015	Extended BCPR activities 2016 onwards
Population register component	Study area:	Study area:
	1. Shahjadpur subdistrict	1. Shahjadpur subdistrict
		2. Other areas with known population denominator and confirming maximum case ascertainment (depending on funding availability)
	Methods:	Methods:
	1. Re-tracing existing CP cohort	1. Active surveillance and screening using KIM
	2. Active surveillance and screening using KIM	
Other registrants		Study area:
		1. Cases available from other research groups, hospitals, NGOs and health care professionals and entered into the register through the BCPR web portal
		Methods:
		1. Opportunistic/purposive recruitment

### Data collection, quality assurance and analysis plan

The standard BCPR record form (i.e., data collection form) has been developed in consultation with international experts. Once the BCPR record form is completed, after assessment by a research physician, data will be entered electronically into the BCPR online data repository. Only the study investigators and nominated delegates will have access to identifiable data. BCPR online data repository is password protected and only individuals with user access would be able to enter any new data. The data coordinating and analysis center is located at the Child Sight Foundation (CSF), in Dhaka, Bangladesh. Internal data quality and data entry error checks will be performed on a routine basis in the data coordinating and analysis centre. The completeness of ascertainment will also be checked through specific studies in future and also by capture recapture method.

Descriptive epidemiological measures, such as prevalence and incidence of CP, will be estimated from the BCPR registry data. We will use Bangladesh Bureau of Statistics (BBS) most recent census data to calculate the denominator population. Frequencies of different types of CP will be presented in percentage with 95 % confidence interval (95 % CI).

### Ethical considerations

#### Ethics approval

The study has been approved by the Cerebral Palsy Alliance NHRMC Human Research Ethics Committee (Ref no. 2015-03-02) in Australia and the Asian Institute of Disability and Development (AIDD) Human Research ethics committee (southasia-irb-2014-l-01) and Bangladesh Medical Research Council (BMRC) HREC (BMRC/NREC/2013-2016/1267) in Bangladesh.

#### Informed consent

Initial contact with families of children with CP would be made through the local ‘key informants’ and health professionals. Families will be provided with information about the CP Register. The health professionals will explain in detail the purpose of the CP Register. If they are illiterate, the information sheet will be read out to the parents in local language (Bengali) and written consent will be obtained by thumbprint from the parents.

#### Confidentiality and privacy

The coded non-identifiable BCPR data will be stored on a secure server maintained by the Cerebral Palsy Alliance in Australia. The BCPR dataset will be accessible by the administrator only, with computers protected by secure password log-on instigated after 5 minutes of computer inactivity. An appropriate data backup schedule is already in place. No data is stored on any researcher’s local computer environment. No identifiable information will be presented in any reports nor made apparent through detail of specific personal or health characteristics. A non-identifiable dataset will be shared with the Australian Cerebral Palsy Register to enable international comparisons.

### Implementation, baseline study characteristics and evaluation

The BCPR online data repository has been developed and tested. It will soon become operational and data from the paper-based BCPR record forms will be entered into the online data repository. A BCPR advisory board has been formed in Bangladesh and a data sharing agreement has been signed between Child Sight Foundation (CSF), Bangladesh and the Cerebral Palsy Alliance Research Institute, Australia.

Following ethics approval in both Bangladesh and Australia we commenced BCPR study activities from January 2015. To date we have recruited 299 children with CP into the register. Their median age was 8.5 years and median age of diagnosis of CP was 4 years. Recruitment into the pilot study will continue till December 2015.

Once the pilot study is complete (i.e., Dec 2015), we will evaluate the feasibility of BCPR focusing on the scientific (e.g., recruitment rates, participant flow, retention rates), budget and management (e.g., human and data optimization) issues [21].

## Discussion

Once implemented, BCPR will be, to the best of our knowledge, the first CP register in a LMIC. Major strengths of this project are; The use of established infrastructure form the Australian Cerebral Palsy Register and technical support from the Cerebral Palsy Alliance; plus the support and implementation by staff of CSF, Bangladesh. CSF has long-standing track record in working with children with disabilities at the community level. We have also applied innovative and cost-effective approaches (e.g., Key Informants Method) for case ascertainment in the community.

We are expecting to disseminate results by early 2016. The BCPR will have a number of immediate social and public health benefits : 1) We will be identifying children with CP from rural communities in Bangladesh and facilitate rehabilitative services; 2) the CP register will give a precise estimate of incidence, prevalence and profile of children with CP and, the aetiology and risk factors for CP in Bangladesh; and 3) The CP register could also be used as a sampling frame for future research e.g., to do intervention trials that evaluate cost-effective intervention strategies to promote functional abilities and limit secondary impairments in children with CP.

## Abbreviations

CP: Cerebral palsy
KIM: Key informants method
ACPR: Australian cerebral palsy register
BCPR: Bangladesh cerebral palsy register
NGO: Non-government organisation
CSF: Child sight foundation
BBS: Bangladesh bureau of statistics
AIDD: Asian institute of disability and development
BMRC: Bangladesh medical research council
HREC: Human research ethics committee
